# Supplementary material for: Dissecting the genetic architecture of knee alignment reveals its contribution to osteoarthritis risk
Source: medRxiv. 2026 Jun 25:2026.06.23.26356332. Preprint. [Version 1] doi: 10.64898/2026.06.23.26356332 (PMC13321165; doi:10.64898/2026.06.23.26356332)
Supplement: Supplementary file 1 [file NIHPP2026.06.23.26356332V1-supplement-1.pdf]

medRxiv preprint doi: https://doi.org/10.64898/2026.06.23.26356332; this version posted June 25, 2026. The copyright holder for this preprint (which was not certified by peer review) is the author/funder, who has granted medRxiv a license to display the preprint in perpetuity.
It is made available under a CC-BY 4.0 International license .

Supplementary methods

Population

UK Biobank is a prospective cohort that recruited 503,317 individuals aged 40–69 between 2006-10. It collected extensive genetic, questionnaire, clinical assessment and linked electronic health record data on each participant. Since 2014, a subset of participants has undergone high-resolution dual-energy X-ray absorptiometry (DXA) imaging of both knees and hips using a GE-Lunar iDXA scanner (Madison, WI) [1]. UK Biobank received ethical approval from the North-West Multi-Centre Research Ethics Committee (11/NW/0382). All participants provided informed consent, including linkage to health records. This project was approved under UK Biobank application 17295.

Manual validation of automated femorotibial

Model performance was evaluated against manual annotations in 100 randomly selected scans using concordance correlation coefficients (CCC), demonstrating excellent agreements between manual and automated annotations (CCC 0.95; 95% CI 0.92–0.96). Reliability was assessed in the same 100 scans using intraclass correlation coefficients based on the three repeated measurements, demonstrating excellent repeatability for both manual and automated measurements (Supplementary Table 1).

Observational analyses

The control group for the observational analyses consisted of participants without either varus or valgus deformities. That is the control group had neutral alignment.

Time to event analyses were restricted to participants with DXA imaging acquired before the study end date [30th October 2022], which was the last date of electronic healthcare linkage. They were censored on death. Analysis was adjusted ethnicity categorized as White versus non-White due to the low numbers of non-White participants.

GWAS

Genotyping, imputation and quality control (QC) were performed by UKB as previously described [2]. Samples were genotyped using two genotyping arrays; Applied Biosystems UK BiLEVE Axiom Array by Affymetrix (49,950 participants) and Applied Biosystems UK Biobank Axiom Array (438,427 participants). Data were imputed using the HRC reference panel, and the merged UK10K and 1000 Genomes phase 3 reference panels in IMPUTE4.

medRxiv preprint doi: https://doi.org/10.64898/2026.06.23.26356332; this version posted June 25, 2026. The copyright holder for this preprint (which was not certified by peer review) is the author/funder, who has granted medRxiv a license to display the preprint in perpetuity.
It is made available under a CC-BY 4.0 International license .

Ancestry assignment of UKB participants was performed as follows: the UKB sample was projected onto the first 20 principal components estimated from the 1000 Genomes Phase 3 (1000G) project (where ancestry was known) using GCTA version 1.93.2. Projections used a curated set of 38,512 LD-pruned HapMap 3 Release 3 (HM3) bi-allelic SNPs that were shared between the 1000G and UKB genotyped datasets (i.e. MAF > 1%, minor allele count > 5, genotyping call rate > 95%, Hardy-Weinberg P > $1x10^{-6}$, and regions of extensive LD removed). Uniform Manifold Approximation and Projection for Dimension Reduction (UMAP) was used in conjunction with the first 20 principal components to cluster 486,445 individuals using the following parameters: min_dist=0.0001, n_components=3, n_neighbors=45, random_state=10293082. UKB participants that clustered together with the 1000G European sub-populations were manually identified by visual inspection (N=461,920) and used for downstream genetic analyses.

Genetic correlation - traits

Osteoarthritis phenotypes including hip osteoarthritis, total hip replacement, knee osteoarthritis, and total knee replacement, were obtained from the largest osteoarthritis GWAS [3]. Height and body mass index were obtained from GIANT consortium [4, 5]. Weight, whole-body lean mass, appendicular lean mass, femoral neck width, and tibio-femoral angle were derived from genome-wide association studies conducted in the UK Biobank [6-9]. Femoral neck bone mineral density (FN-BMD) and total-body DXA-derived BMD were obtained from previous research [10, 11].

Functional genetic data

Colocalization

Expression quantitative trait loci (eQTL) colocalization analyses were performed between the associated loci and eQTL signal to identify regulatory variants influencing gene expression. eQTL data were derived from adult joint tissues, including macroscopically highly degraded and low-degraded cartilage, synovium, and infrapatellar fat pad obtained from individuals undergoing joint replacement surgery [12]. Methylation quantitative trait loci (mQTL) colocalization analyses were also conducted to evaluate genetic effects on DNA methylation at CpG sites. mQTL data were obtained from the same adult joint tissues as the eQTL analyses, as well as from fetal knee cartilage samples collected between 7 and 21 post-conception weeks [12, 13]. Evidence of colocalization was considered suggestive where the posterior probability of a shared causal variant (PP4) was ≥0.60 and confirmed where the posterior probability of a

medRxiv preprint doi: https://doi.org/10.64898/2026.06.23.26356332; this version posted June 25, 2026. The copyright holder for this preprint (which was not certified by peer review) is the author/funder, who has granted medRxiv a license to display the preprint in perpetuity.
It is made available under a CC-BY 4.0 International license .

shared causal variant (PP4) was ≥0.80. Closest genes were annotated using CpG-to-gene mapping based on Illumina HumanMethylation450K array annotation (hg19), with rsID positions aligned to GRCh37. Where no gene annotation was available, the nearest gene was identified manually based on base-pair position on the chromosome using dbSNP [14].

ATAC-seq

Chromatin accessibility was assessed using assay for transposase-accessible chromatin sequencing (ATAC-seq) in fetal skeletal tissues (67 days post-fertilization), fetal knee cartilage (12 post-conception weeks), and adult talar cartilage samples. The closest genes were annotated based on chromosomal base-pair position using dbSNP [14-17].

Generalized gene-set analysis

To explore potential links between femorotibial angle-associated genes and bone cell function, we performed MAGMA competitive gene set analyses on single-cell transcriptomics data generated from human femoral heads, which primarily consist of trabecular bone. Firstly, gene-based association tests were conducted using MAGMA software (v1.10) [18] applied to the European femorotibial angle GWAS summary statistics. Patterns of linkage disequilibrium were modelled with a reference sample of ~50,000 unrelated individuals of European ancestry from the UK-Biobank Study[19]. Genetic variants were annotated to protein-coding genes based on Ensembl gene version GRCh37. A variant was assigned to a gene if it was located within 2 kb upstream of the transcription start site or within 1 kb downstream of the transcription end site. A multi-model approach that combined results from two complementary gene-level association models, was applied: the ‘SNP-wise mean’ model that calculates the mean of $\chi 2$ statistics for all genetic variants annotated to a gene, as well as the ‘SNP-wise top’ model that calculates the $\chi 2$ statistic for the lead genetic variant annotated to a gene. The aggregated p-value from this approach (p_{Multi}) reflects the overall strength of evidence for association between each protein coding gene and femorotibial angle. The threshold to declare statistical significance was determined using *Bonferroni* correction as follows: error rate of one test / number of genes tested = 0.05 / 20 = $p < 2.5\times10^{-3}$.

Human single cell RNA sequencing data was obtained from previously published work[19]. In brief, femoral heads were collected from patients undergoing joint replacement surgery. Bone cores were excised and digested. Viable human cells ($DAPI^{-}$) negative for erythroid marker CD235a were sorted into CD45+ (haematopoietic) and $CD45^{-}$ (non-haematopoietic)

medRxiv preprint doi: https://doi.org/10.64898/2026.06.23.26356332; this version posted June 25, 2026. The copyright holder for this preprint (which was not certified by peer review) is the author/funder, who has granted medRxiv a license to display the preprint in perpetuity.
It is made available under a CC-BY 4.0 International license .

populations. Cells were processed using the 10x Chromium platform and Chromium Single Cell 3' v2 Reagent Kit (target 10,000 cells/channel), followed by sequencing on the Illumina NovaSeq 6000 Sequencing System. Raw data were processed with CellRanger (v2.7) and analysed using the Seurat package (v4 and v5)[20]. Log normalisation was applied and low-quality cells filtered (fewer than 300 genes or Mitochondrial Unique Molecular Identifier (UMI) fraction>10%). Dimensionality reduction was performed using 20 principal components derived from the top 3,000 variable genes, and batch correction was applied using Harmony[21]. Clustering was performed using the Louvain Method on shared nearest neighbour graphs and visualised by Uniform Manifold Approximation and Projection (UMAP). Cell types were annotated using canonical markers and cluster-specific gene programs as described in Chai et al[19]. Differentially expressed genes (gene programs) were identified using Seurat FindAllMarkers, requiring log2FC>0.5 and Bonferroni-adjusted $p<0.05$.

Evidence of enrichment was inferred by testing whether genes comprising each cell-type gene program were more strongly associated with femorotibial angle on average than protein-coding genes not included in the program. Gene set analysis (GSA) used data from gene-based tests of association and accounted for several confounding factors including gene size, gene density, and the inverse of the mean minor allele count in the gene, as well as the log value of these three factors. Post-hoc permutation analysis was conducted on enriched gene-programs using R-scripts supplied with the MAGMA software using default settings[22].

RNA-seq

Gene expression was evaluated using bulk RNA sequencing (RNA-seq) data from adult musculoskeletal tissues, including multiple tendon-related tissues (hamstring, posterior tibialis, extensor hallucis longus, Achilles, patellar, quadriceps, supraspinatus, and long head of biceps tendons). Expression levels were quantified as $\log_2$counts per million ($\log_2$CPM), and genes were ranked based on mean expression across tissues. The top 25% of genes were defined as relatively highly expressed, with the top 10% representing a more stringent high-confidence subset, based on relative expression levels within this dataset rather than absolute expression across tissues [23].

medRxiv preprint doi: https://doi.org/10.64898/2026.06.23.26356332; this version posted June 25, 2026. The copyright holder for this preprint (which was not certified by peer review) is the author/funder, who has granted medRxiv a license to display the preprint in perpetuity.
It is made available under a CC-BY 4.0 International license .

References

1. Littlejohns, T.J., et al., *The UK Biobank imaging enhancement of 100,000 participants: rationale, data collection, management and future directions.* Nature Communications, 2020. **11**(1): p. 2624.
2. Bycroft, C., et al., *The UK Biobank resource with deep phenotyping and genomic data.* Nature, 2018. **562**(7726): p. 203-209.
3. Boer, C.G., et al., *Deciphering osteoarthritis genetics across 826,690 individuals from 9 populations.* Cell, 2021. **184**(24): p. 6003-6005.
4. Yengo, L., et al., *Meta-analysis of genome-wide association studies for height and body mass index in ∼700000 individuals of European ancestry.* Hum Mol Genet, 2018. **27**(20): p. 3641-3649.
5. Yengo, L., et al., *A saturated map of common genetic variants associated with human height.* Nature, 2022. **610**(7933): p. 704-712.
6. Venkatesh, S.S., et al., *Characterising the genetic architecture of changes in adiposity during adulthood using electronic health records.* Nat Commun, 2024. **15**(1): p. 5801.
7. Pei, Y.F., et al., *The genetic architecture of appendicular lean mass characterized by association analysis in the UK Biobank study.* Commun Biol, 2020. **3**(1): p. 608.
8. Tobias, J.H., et al., *Femoral neck width genetic risk score is a novel independent risk factor for hip fractures.* J Bone Miner Res, 2024. **39**(3): p. 241-251.
9. Kun, E., et al., *The genetic architecture and evolution of the human skeletal form.* Science, 2023. **381**(6655): p. eadf8009.
10. Zheng, H.F., et al., *Whole-genome sequencing identifies EN1 as a determinant of bone density and fracture.* Nature, 2015. **526**(7571): p. 112-7.
11. Medina-Gomez, C., et al., *Life-Course Genome-wide Association Study Meta-analysis of Total Body BMD and Assessment of Age-Specific Effects.* Am J Hum Genet, 2018. **102**(1): p. 88-102.
12. Katsoula, G., et al., *Primary cartilage transcriptional signatures reflect cell-type-specific molecular pathways underpinning osteoarthritis.* Am J Hum Genet, 2024. **111**(12): p. 2735-2755.
13. McDonnell, E., et al., *The methylomic landscape of human articular cartilage development contains epigenetic signatures of osteoarthritis risk.* The American Journal of Human Genetics, 2024. **111**(12): p. 2756-2772.
14. Sherry, S.T., et al., *dbSNP: the NCBI database of genetic variation.* Nucleic Acids Res, 2001. **29**(1): p. 308-11.
15. Rice, S.J., et al., *Genetic risk of osteoarthritis operates during human skeletogenesis.* Human Molecular Genetics, 2022. **32**(13): p. 2124-2138.
16. Richard, D., et al., *Functional genomics of human skeletal development and the patterning of height heritability.* Cell, 2025. **188**(1): p. 15-32.e24.
17. Kramer, N.E., et al., *Response eQTLs, chromatin accessibility, and 3D chromatin structure in chondrocytes provide mechanistic insight into osteoarthritis risk.* Cell Genomics, 2025. **5**(1): p. 100738.
18. de Leeuw, C.A., et al., *MAGMA: generalized gene-set analysis of GWAS data.* PLoS Comput Biol, 2015. **11**(4): p. e1004219.

medRxiv preprint doi: https://doi.org/10.64898/2026.06.23.26356332; this version posted June 25, 2026. The copyright holder for this preprint (which was not certified by peer review) is the author/funder, who has granted medRxiv a license to display the preprint in perpetuity.
It is made available under a CC-BY 4.0 International license .

19. Chai, R.C., et al., *Multiscale analysis and functional validation of the cellular and genetic determinants of skeletal disease.* bioRxiv, 2024.
20. Hao, Y., et al., *Integrated analysis of multimodal single-cell data.* Cell, 2021. **184**(13): p. 3573-3587.e29.
21. Korsunsky, I., et al., *Fast, sensitive and accurate integration of single-cell data with Harmony.* Nature Methods, 2019. **16**(12): p. 1289-1296.
22. de Leeuw, C.A., et al., *Conditional and interaction gene-set analysis reveals novel functional pathways for blood pressure.* Nat Commun, 2018. **9**(1): p. 3768.
23. Mimpen, J.Y., et al., *Exploring cellular changes in ruptured human quadriceps tendons at single-cell resolution.* J Physiol, 2025. **603**(16): p. 4535-4554.
